# Carbene modification and reversible crosslinking of silver nanoparticles for controlled antibacterial activity

**DOI:** 10.1038/s41598-020-72043-1

**Published:** 2020-09-10

**Authors:** Liling Jing, Mark G. Moloney, Hao Xu, Lian Liu, Wenqiang Sun, Junying Li, Pengfei Yang

**Affiliations:** 1 School of Chemistry and Pharmaceutical Engineering, Qilu University of Technology (Shandong Academy of Sciences), Jinan, 250353 People’s Republic of China; 2 Chemistry Research Laboratory, Department of Chemistry, University of Oxford, Oxford, OX1 3TA UK; 3 Oxford Suzhou Centre for Advanced Research, Suzhou, 215123 People’s Republic of China

**Keywords:** Chemical modification, Surface chemistry, Biomedical materials

## Abstract

Silver nanoparticles (Ag NPs) system capable of exhibiting different particle size at different temperature was developed, which depended on the extent of Diels–Alder (DA) reaction of bismaleimide with furan. Thus, Ag NPs were functionalized on the surface by a furyl-substituted carbene through an insertion reaction. Subsequent reversible DA crosslinking achieved a controlled aggregation with different particle size, which gives a series of different antibacterial activity. These Ag NPs were characterized by Scanning Electron Microscopy (SEM), Transmission Electron Microscopy (TEM), X-ray Photoelectron Spectroscopy (XPS), and Nanoparticle Size Analyzer. The aggregation of the Ag NPs could be reliably adjusted by varying the temperature of DA/reverse-DA reaction. The antibacterial activity was assessed using the inhibition zone method against *Escherichia coli* (*E. coli*) and *Staphylococcus aureus* (*S. aureus*), which decreased first and then increased in agreement with the size evolution of Ag NPs. This approach opens a new horizon for the carbene chemistry to modify silver nanoparticles with variable size and give controlled antibacterial activity.

## Introduction

In recent decades, silver nanoparticles (Ag NPs) have been intensively investigated for their unique physical, chemical, and biological properties which underpin applications in various fields, including antibacterial devices^1–3^, electric coatings^4,5^, sensors^6,7^, and catalysts^8,9^. It is especially noteworthy that Ag NPs are an important type of broad-spectrum antibacterial materials that show excellent activity against almost all microorganisms; this activity appears to derive from the large surface area which can generate Ag^+^ much faster than bulk silver metal, allowing wide application in medical science^10–12^.

However, nanotechnology is a double-edged sword, and it has been found that the antibacterial activity of Ag NPs depends on several parameters, including particle size^13,14^, particle shape^15,16^, surface coating^17,18^, and co-additives^19,20^. The interaction of these factors makes the development of Ag NPs complicated^21^. Most importantly, while their small-size effect gives Ag NPs excellent antibacterial activity, it also endows them with high toxicity to animal cells. Thus, when Anda et al.^22^ investigated the toxicity of Ag NPs to BEAS-2B cells, a type of human lung cells, they showed that small Ag NPs were cytotoxic for human lung cells and the toxicity was associated with the rate of intracellular Ag release, a “Trojan horse” effect, which increased the leakage of cell membrane and lowered cellular activity. On the other hand, Ag NPs with large size (≥ 100 nm) could not enter the interior of BEAS-2B cells so that these Ag NPs showed lower cytotoxicy. However, it is very hard to adjust the bioactivity of silver-contained materials after it is ready to use. That is, it is not easy for Ag NPs to realize controlled drug release that are widely used in the areas of internal treatment^23^, regenerative engineering^24^, and intelligent medicine^25^. For this reason, it is highly attractive to develop a simple and convenient method that can adjust the size of Ag NPs by post-treatment, so as to give high bioactivity and low cytotoxicy when needed.

In this paper, the reverse Diels–Alder reaction between the end-group furans and bismaleimides (BMI) was chosen to adjust the size of nanoparticles. This “click” reaction could introduce the dynamic covalent crosslinking to control the extent of crosslinking, thus to give temperature-dependent structures of already formed Ag NPs, which was expected to obtain a series of Ag NPs aggregates with controlled size and different antibacterial activity. More importantly, this approach could conveniently realize controlled release behavior by post-treatment instead of synthetic procedure, and might have the potential application to adjust the bioactivity of silver-contained materials after it is ready to use.

## Results

### Synthesis of diazomethane

**Scheme 1:**
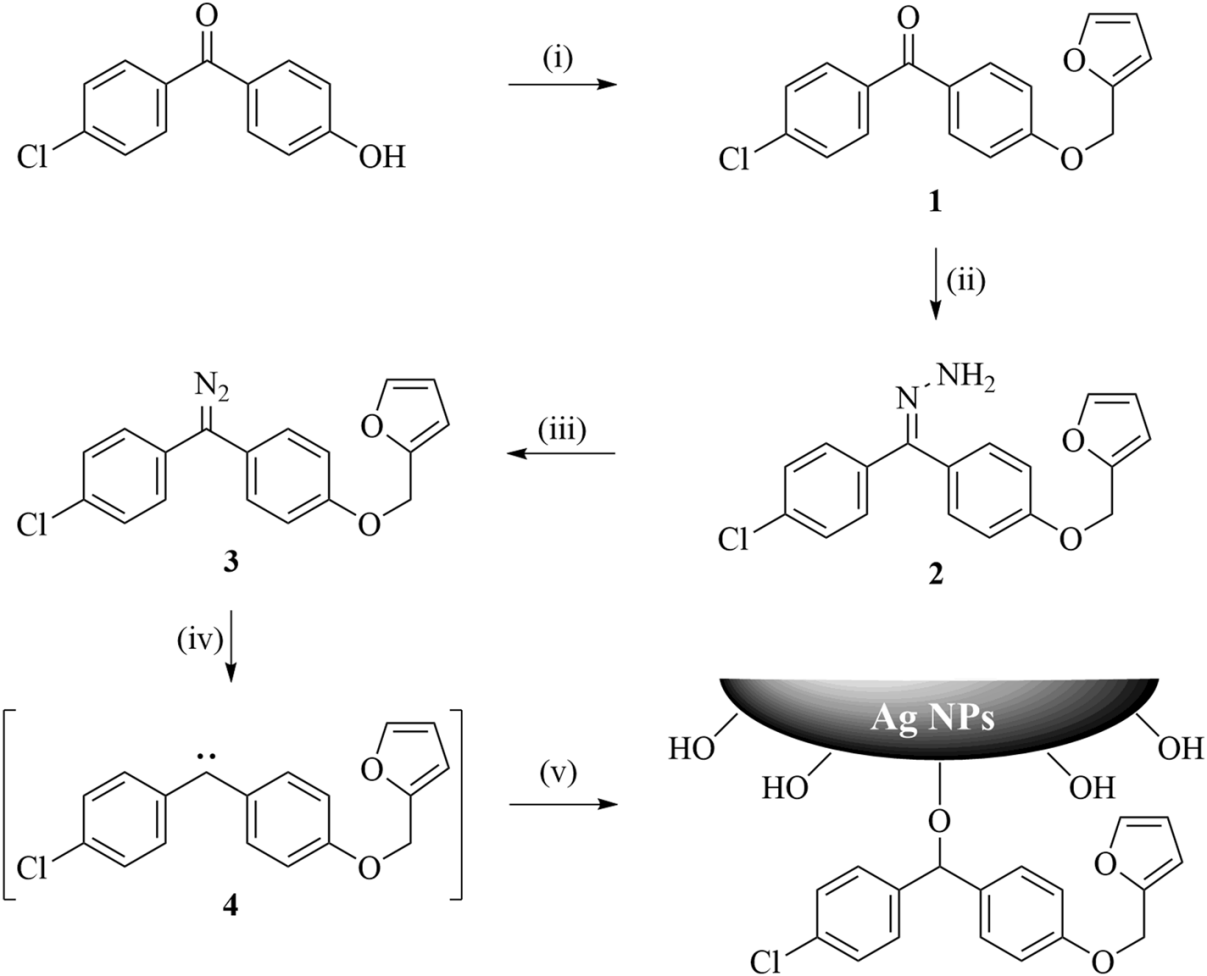
Synthesis of furyl diazomethane and surface modification of Ag NPs. (i) furfuryl alcohol, diethyl azodicarboxylate, triphenylphosphine, THF, 0 °C → rt., 12 h (74%); (ii) NH_2_NH_2_·H_2_O, HAc, EtOH, reflux, 24 h (86%); (iii) MnO_2_, Na_2_SO_4_, KOH, dichloromethane, r.t., 2 h (98%); (iv) 120 °C; (v) Ag NPs.

As shown in Scheme 1, 4-chloro-4′-hydroxybenzophenone was reacted with furfuryl alcohol in the presence of triphenylphosphine and diethyl azodicarboxylate (DEAD) to give furyl-substituted benzophenone **1**, which was subsequently treated with hydrazine monohydrate to generate hydrazone **2** in good yield. The hydrazone was then oxidized with manganese dioxide to obtain furyl diazomethane **3**. 4-Chloro-4′-hydroxybenzophenone is chosen as substrate because the phenolic hydroxyl group provides convenient conditions to permit introduction of the furyl side chain, but unfortunately also activates the diazomethane towards carbene formation; the electron withdrawing chloro group on the other aromatic ring helps to provide some additional stability to the diazomethane^26^. This protocol is designed to introduce furyl groups into the diazomethane by mild reaction conditions. The diazomethane **3** was stable enough for storage, but easily generated furyl carbene **4** and reacted with Ag NPs through the insertion reaction at higher temperature or under solar light. This difference of reactivity was required for its convenient application as a surface modifying agent.

### Preparation of crosslinked Ag NPs

Ag NPs were obtained by the reduction of AgNO_3_ in the presence of glycol, followed by their blending with furyl diazomethane **3** at 120 °C. The furyl carbene **4** was then generated and reacted with functional groups on the surface of Ag NPs by insertion reaction, so as to give furan-modified Ag NPs. This is a rapid and mild approach to modify not only Ag NPs by also other nanomaterials including SiO_2_^27^ and TiO_2_^28^, which could be compared with the traditional reaction of thiol-Ag^29,30^ or thiol-Au^31,32^ modification. Bismaleimide (BMI) was subsequently added to a dispersion of furan-modified Ag NPs in dimethylformamide, and Diels–Alder (DA) crosslink was achieved at different temperature (50 °C, 70 °C, 90 °C, 110 °C, 130 °C).

### Morphology analysis

**Figure 1:**
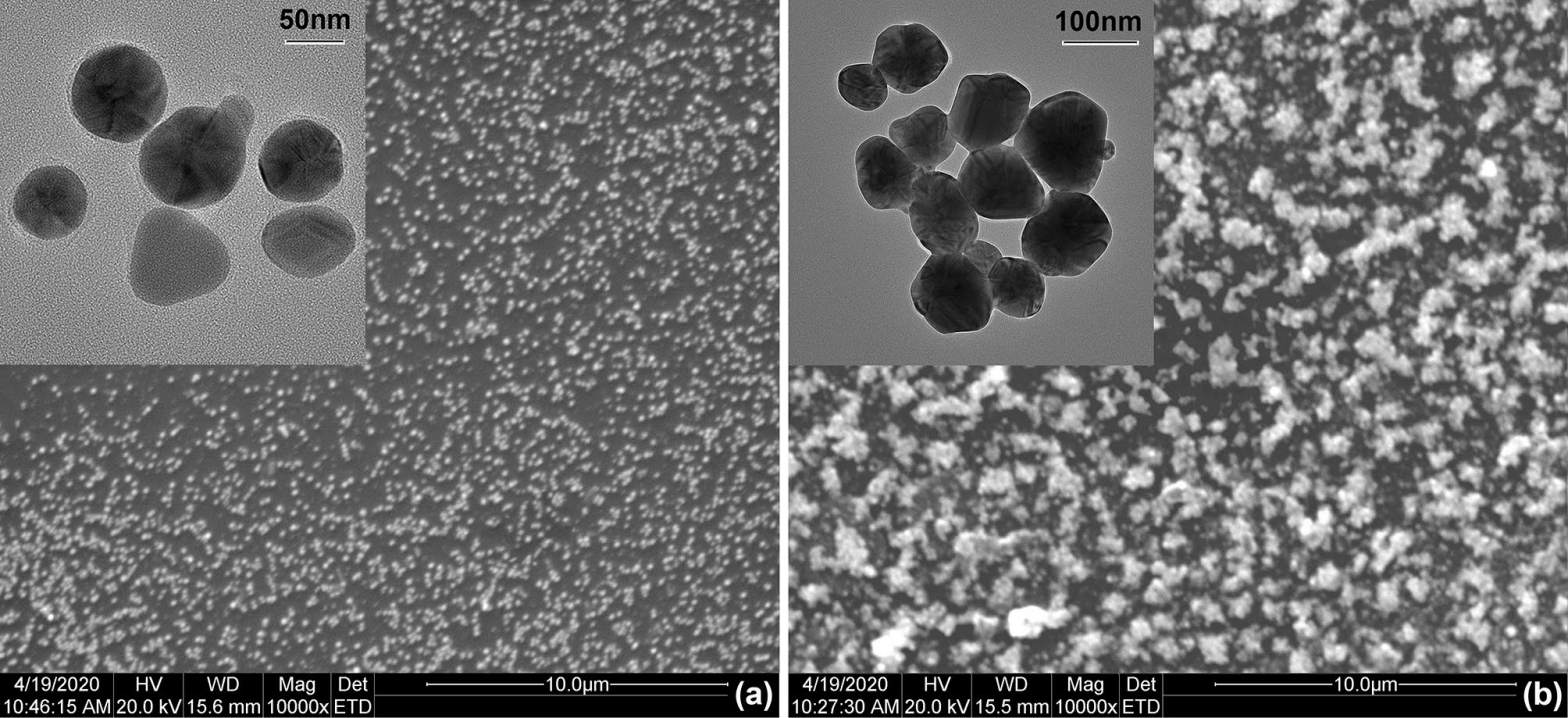
SEM and TEM images of Ag NPs. (**a**) The initial Ag NPs are shown to be uniformly distributed without aggregation. The particle size is 50–70 nm. (**b**) Ag NPs are crosslinked and gather together at 70 °C. The particle size is 280–300 nm. This clearly demonstrates the DA crosslinking between Ag NPs.

An overall impression of Ag NPs before and after crosslink was given by Scanning Electron Microscopy (SEM). The initial Ag NPs were shown to be uniformly distributed without aggregation in Fig. 1a. In contrast, they were gathered together to be larger particles in Fig. 1b, which clearly demonstrated the DA crosslinking between Ag NPs. Transmission Electron Microscopy (TEM) was used to give a more visualized characterization, and the photos were shown in the up-left corner of Fig. 1. The size of Ag NPs varied from 50–70 nm in Fig. 1a to 280–300 nm in Fig. 1b after being reacted with BMI. It was noteworthy that these Ag NPs seemed to be crosslinked with each other, instead of overlap or cumulate. That is, the chemical reaction of DA crosslinking by covalent bonds attached Ag NPs together and no distinct border was found for each neighboring region.

### Particle size analysis

**Figure 2:**
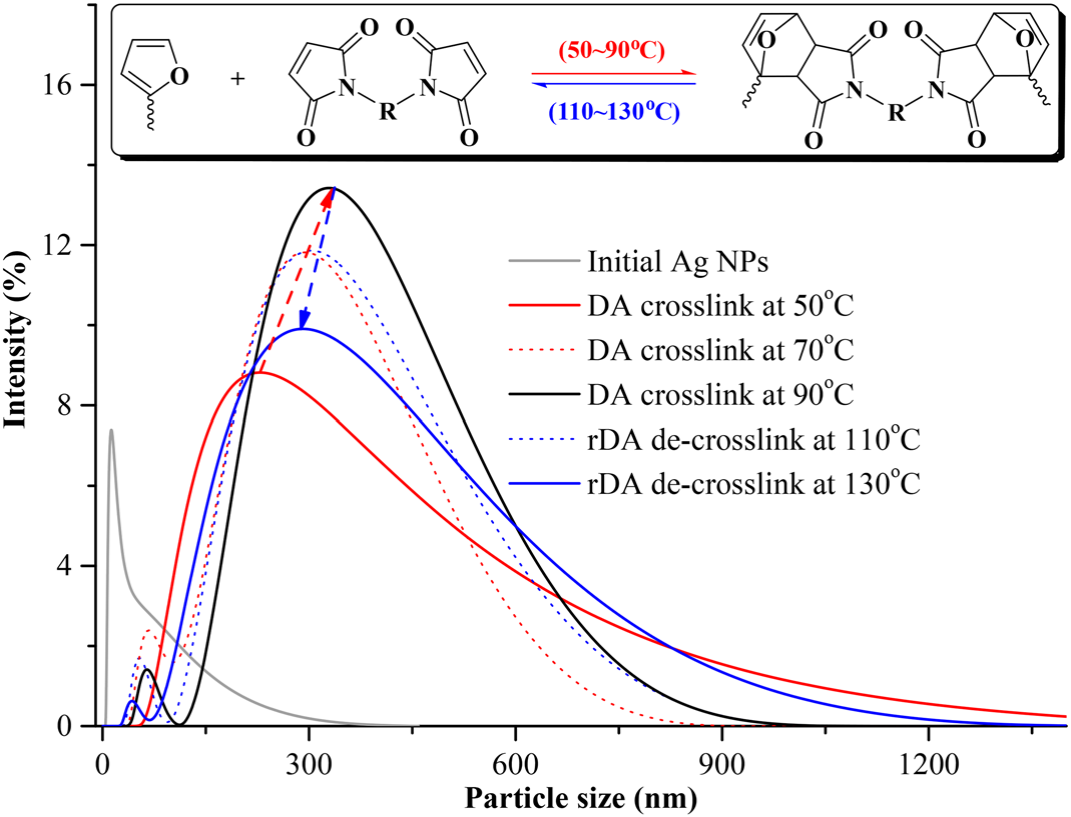
The evolution of Ag NP size during reverse Diels–Alder (DA/rDA) reaction. The grey curve shows the particle distribution of initial Ag NPs. The red arrow from red curve to black curve shows the increase of particle size when the temperature rises from 50 to 70 °C. This demonstrates the success of DA crosslinking. The blue arrow from black curve to blue curve shows the decrease of particle size when the temperature rises from 90 to 130 °C. This demonstrates the success of reverse DA crosslinking.

The DA reaction has been reported to be a temperature-controlled equilibrium, for temperatures ranging from 50 °C to more than 90 °C^33–35^. The surface-modified Ag NPs with furyl groups would react with BMI at relatively low temperature, and the reverse DA reaction might become prominent after more than 90 °C. This speculation was confirmed by particle size analysis. As shown in Fig. 2, the peak value represents the size of different Ag NPs. The initial Ag NPs were obtained with the smallest size of < 100 nm. After carbene modification and DA crosslinking with BMI at different temperature, the particles became enlarged to 220 nm (50 °C), 300 nm (70 °C), and 340 nm (90 °C), and then decreased to 310 nm (110 °C), and 280 nm (130 °C). The reason for this trend was that the DA crosslink proceeded at relatively low temperature (50–90 °C) to make the Ag NPs aggregate, while rDA reaction occurred at a little higher temperature (110–130 °C) to disassociate the Ag NPs. This outcome was in accordance with the observation of SEM and TEM images, which indicated that the DA/rDA reaction equilibrium could be easily used to control the aggregation of Ag NPs just by adjusting reaction temperature, which was proved to be very important for the control of antibacterial activity.

### Surface analysis

**Figure 3:**
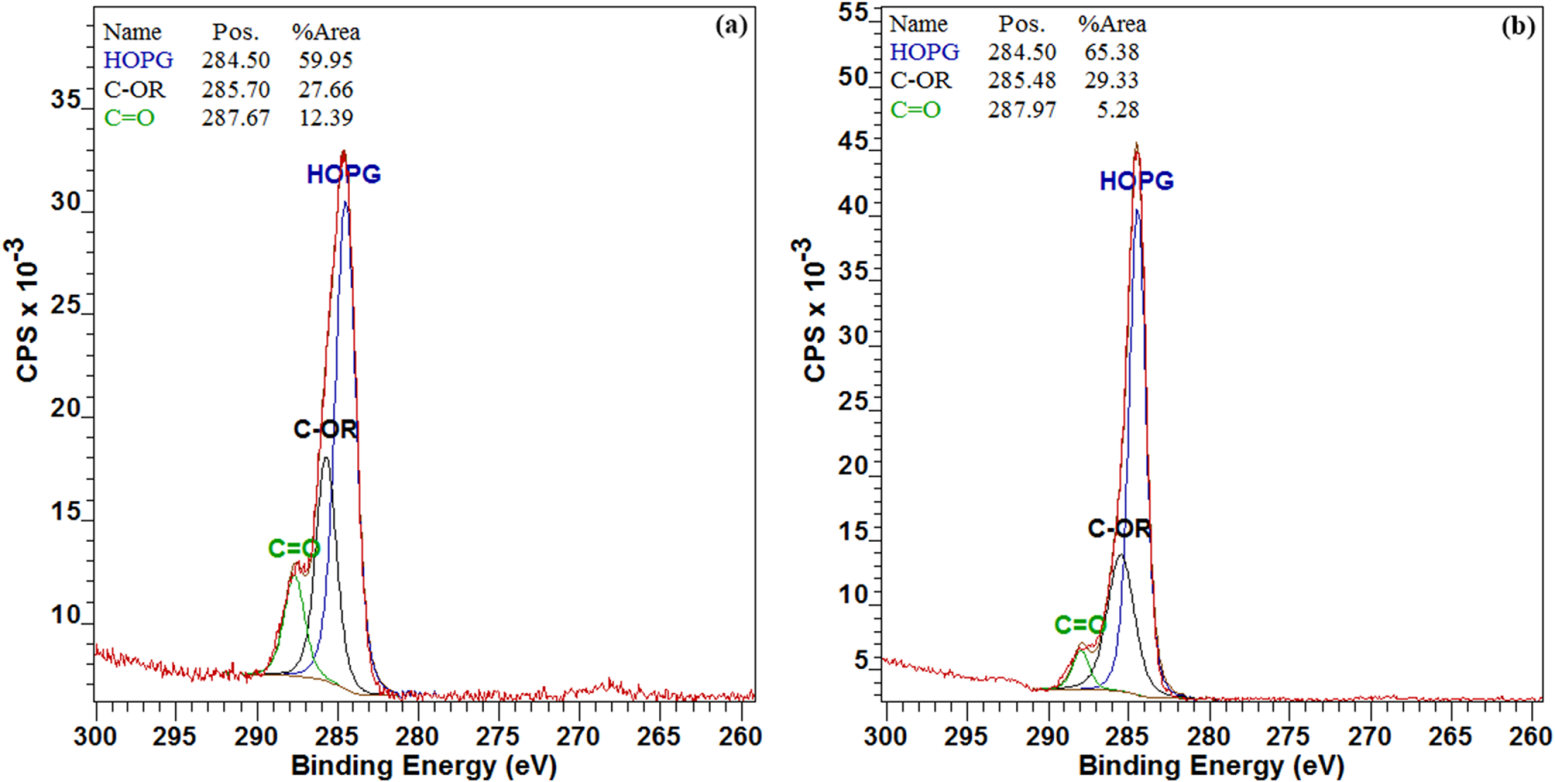
C1s XPS spectra of crosslinked Ag NPs: (**a**) 90 °C; (**b**) 130 °C. When crosslinking temperature changes from 90 to 130 °C, the percentage of C–OR increases and the percentage of C=O decreases. It means that the content of furyl increases and the content of BMI lowers in the crosslinking layer on the surface of Ag NPs.

The chemical component on the surface of Ag NPs would be very different before and after DA crosslinking, since furyl carbene residues would change to furan-BMI adducts after DA reaction. Thus, *x*-ray Photoelectron Spectroscopy (XPS) was used to characterize this variation. Typical Ag NP samples that crosslinked at 90 °C and 130 °C were chosen as examples, and their C 1*s* spectra of XPS were shown in Fig. 3. There are three types of carbon in the spectra: HOPG (284.5 eV), C–OR (285.4–285.7 eV), and C=O (287.6–288.0 eV). C–OR groups and C=O groups are derived from furan and BMI, respectively. These two peaks give evidence that BMI entered into the surface layer of Ag NPs and reacted with furyl groups. When crosslinking temperature changed from 90 to 130 °C, the percentage of C–OR varied from 27.66 to 29.33%, and the percentage of C=O changed from 12.39 to 5.28%. It means that the content of furyl increases and the content of BMI lowers in the crosslinking layer, which is consistent with occurrence of the rDA reaction which liberates the BMI molecules and removes them from the Ag NPs system. This data clearly indicated the success of the temperature-controlled DA/rDA reaction.

### Bioassay

Diverse particle size was achieved when the DA/rDA crosslinking was conducted at different temperature, and the particle size of Ag NPs would be expected to influence their antibacterial activity. For Ag NPs samples of the same weight, a smaller particle has a larger surface area, and releases the silver ion more quickly, so as to give enhanced antibacterial activity. Two bacteria, *Escherichia coli* (*E. coli*) and *Staphylococcus aureus* (*S. aureus*), were chosen for bioassay to characterize all the crosslinked Ag NPs, as well as the non-crosslinked Ag NPs for comparison. Their inhibition zone size was shown in Table 1, and the typical photos of agar plate with inhibition zones were shown in Figure S4–S5.

**Table 1:** The inhibition size of different Ag NPs samples.

Samples	Inhibition zone size (mm)	
	*E. coli*	*S. aureus*
Water	10.0	10.0
Non-crosslinked Ag NPs	14.9	16.2
50 °C-crosslinked Ag NPs	12.6	14.0
70 °C-crosslinked Ag NPs	11.7	13.2
90 °C-crosslinked Ag NPs	10.7	12.7
110 °C-crosslinked Ag NPs	11.3	13.0
130 °C-crosslinked Ag NPs	12.0	13.4

Distilled water is used as a reference. The inhibition zone size decreases first and then increases, which means the antibacterial activity varies in the same order. It means that the Ag NPs enlarge first and then reduce with the rise of crosslinking temperature.

As expected, the non-crosslinked Ag NPs showed the strongest antibacterial activity, which was lowered after being crosslinked. Moreover, when the temperature increased from 50 to 90 °C, the inhibition zone size became smaller. This indicated that the antibacterial activity decreased slowly since Ag NPs increasingly crosslinked through DA reaction. Afterwards, the antibacterial activity began to increase at 110 °C and 130 °C because the rDA reaction disassociated Ag NPs slowly but surely. This was in accordance with the particle size behavior described above.

**Figure 4:**
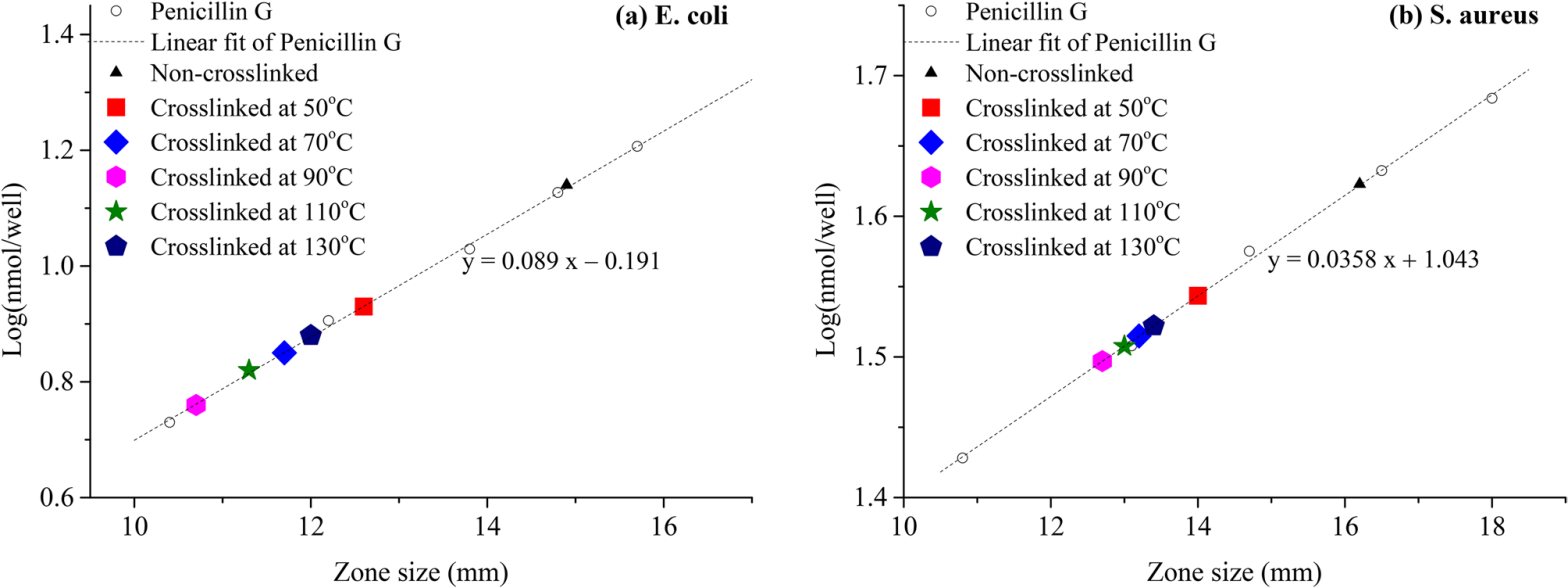
Antibacterial activity of different Ag NPs with penicillin G as calibration. The dotted line means the calibration of penicillin G. The colored symbols represents various Ag NPs samples that crosslinked at different temperature.

In order to compare the antibacterial activity of these Ag NPs with other antibacterial materials^32,35^, penicillin G was used to calibrate the inhibition zone size. As known to all, the diameter of inhibition zone (*D*) is positively related to the amount of antibiotic (*n*), and their linear relationship can be written as Eq. (1):

1 $$\mathrm{Log }\left(n\right)=k\cdot D+b$$

where *n* is the amount of penicillin G (nmol) in the well, *D* is the inhibition zone size (mm), *k* and *b* are constants related to the linear fitting. Thus, the calibration lines of penicillin G for *E. coli* and *S. aureus* were shown in Fig. 4, as well as the fitting equation. Since the inhibition zone size of Ag NPs samples was known (Table 1), their molar equivalents of penicillin G could be calculated according to the fitting equation, thereby depicting their points in Fig. 4. The results would be very useful for preparing silver antibacterial materials with various antibacterial activities.

## Discussion

We have formerly reported a temperature-controlled Ag NP system that relies on the thiophilicity of Ag^+^ for its controlled-release^36^. However, the inevitable binding of Ag^+^ with the thiol in that system would lower the free Ag^+^ from the surface of Ag NPs and reduce the antibacterial activity. The antibacterial mechanism of Ag NPs crucially derives from the fact that Ag^+^ on the surface of Ag NPs can bind thiol groups in enzymes, such as NADH dehydrogenase, thereby disrupting the respiratory chain of bacteria by generating reactive oxygen species, and eventually lead to oxidative stress and cell damage^37–39^. Moreover, since this strategy critically required on the known thiophilicity of Ag NPs, it was not easily generalized for application to a wider range of alternative substrates. To this end, the reverse Diels–Alder reaction between the end-group furans and bismaleimides (BMI) was chosen in this study.

Furyl carbenes, which were generated from furyl diaryldiazomethane by heating, were developed in this paper to surface modify Ag NPs that were prepared by reduction using glycol. The mechanism of carbene modification, which has already been applied for payload delivery^40^ and biological properties^41,42^, lies in the insertion reaction of carbene with X–H bonds (X = C,N,O,…) on the surface of substrates, which would help to reduce the association leading to Ag–S complex formation and ensure the antibacterial activity of Ag NPs.

Ag NPs with the size of 50–70 nm were prepared and functionalized with furyl-substitution on their surface using a carbene insertion reaction. A subsequent crosslink using DA/rDA reaction made Ag NPs aggregate with different size. As known to all, both chemical crosslinking and physical gathering might change the size of Ag NPs aggregate. Since chemical crosslinking was mainly focused on, the influence of physical gathering should be avoided as far as possible. Thus, before characterization, Ag NPs was dispersed in the corresponding solvent by long-time ultrasonic until the dispersion became relatively stable. The time of dispersion was 1.5 h, and all the Ag NPs samples were treated at the same time.

The size of these aggregates was in the range of 220 nm to 340 nm, which could be adjusted by reaction temperature. This controlled aggregation was observed by SEM and TEM clearly, and further confirmed by C 1*s* XPS spectra. It was shown that the particle size greatly increased after the crosslink process was conducted at 50–90 °C, but slightly decreased in the range of 110–130 °C, and this is due to the rDA reaction which disassociated Ag NP aggregates at more than 90 °C. The antibacterial activity of these Ag NPs was investigated, which was shown to be downward first and then upward with the increase of crosslinking temperature, and the turning point was 90 °C. This was in accordance with the evolution of particle size.

The discovery should open a new horizon for preparing Ag NPs to give controlled antibacterial activity. In addition, this strategy could be used for Ag NPs, and therefore potentially a bigger range of substrates, which might have other applications and interest broad researchers in materials science.

## Methods

### Chemicals and instruments

Silver nitrate (BioXtra, > 99%), polyvinylpyrrolidone (BioXtra, K = 30) (PVP), glycol (BioUltra, ≥ 99.5%), triphenylphosphine (ReagentPlus, 99%), diethyl azodicarboxylate (40 wt% in toluene) (DEAD), furfuryl alcohol (reagent grade, 98%), hydrazine monohydrate (reagent grade, 98%), acetic acid (ReagentPlus, ≥ 99%), manganese dioxide (activated, ~ 85%), 4-chloro-4′-hydroxybenzophenone (reagent grade, 98%), *N*,*N*′-(4,4′-methylenediphenyl) dimaleimide (95%) (BMI) were purchased from Sigma-Aldrich (Saint Louis, Missouri). Other solvents (analytical reagent, > 99%) and inorganic salts (analytical reagent, > 99%) were purchased from Sinopharm Chemical Reagent Co. Ltd (Shanghai, China). Two bacterial strains, *E. coli* DH 5α and *S. aureus* Wood 46, were bought from J&K Scientific (Beijing, China). All the chemicals and materials were used as received without further purification.

SEM photographs were taken on a Quanta scanning electron microscope (FEI Co., Oregon) after the samples were coated with gold (~ 20 nm thickness). TEM photographs were taken on a JEM-2100F field emission electron microscope manufactured by JEOL (Tokyo, Japan). XPS analysis was performed with a EscalabXi + X-ray photoelectron spectrometer (Thermo Fisher Scientific, UK) with a reference of 284.5 eV (C 1*s*). Particle size and its distribution were analyzed by a Zetasizer Nano ZS90 laser particle size analyzer produced by Malvern Panalytical (Malvern, UK).

### Synthesis of 4-chloro-4′-furfuryloxybenzophenone

Triphenylphosphine (11.8 g, 45.0 mmol) was dissolved in dried tetrahydrofuran (40.0 ml). To this was added DEAD in toluene solution (19.6 g, 45.0 mmol) and the mixture was stirred for 30 min at 0 °C. After 4-chloro-4′-hydroxybenzophenone (7.00 g, 30.1 mmol) was added to the solution, furfuryl alcohol (2.60 ml, 29.8 mmol) was added drop wise. The mixture was stirred for 12 h. Tetrahydrofuran was then evaporated and dichloromethane (75 ml) was added. The organic phase was washed with H_2_O_2_ aq. solution (40%, 25 ml × 3), saturated NaCl aq. solution (25 ml × 3), dried with MgSO_4_, and concentrated under reduced pressure. The crude product was purified by chromatography on a silica gel column eluting with *n*-hexane/ethyl acetate (8:1), or dissolved in ethyl acetate (3 ml) and precipitated in n-hexane (25 ml) to give the product **1** as a white solid (74%). m.p.: 129.4–130.7 °C; δH (400.3 MHz, CDCl_3_): 5.11 (s, 2H), 6.44 (m, 2H), 7.07 (d, 2H), 7.47 (m, 3H), 7.72 (d, 2H), 7.81 (d, 2H); δC (100.7 MHz, CDCl_3_): 62.5, 110.4, 110.6, 114.5, 128.5, 130.3, 131.1, 132.4, 136.5, 138.3, 143.3, 149.5, 162.1, 194.1; IR ν_max_ (cm^−1^): 2,929 (w), 1,638 (m), 1,599 (m), 1,250 (s), 837(s), 757(vs); HRMS: m/z calcd for C_18_H_14_ClO_3_: 313.0621; found: 313.0623 [M + H]^+^.

### Synthesis of 4-chloro-4′-furfuryloxybenzophenone hydrazone

According to the references^42,43^, a suspension of **1** (2.00 g, 6.39 mmol) in ethanol (80 ml) was treated with hydrazine monohydrate (3.19 ml, 63.9 mmol). A few drops of acetic acid (about 0.5 ml) were added as catalyst. The resulting mixture was heated to a gentle reflux for 96 h. After cooling, the solution was evaporated under vacuum. The residue was dissolved in dichloromethane (100 ml), washed with water (50 ml × 4), dried with MgSO_4_, and evaporated under vacuum. The crude product **2** (86%) of a light yellow liquid as a mixture of diastereoisomers was used without further purification. δH (400.3 MHz, CDCl_3_): 5.01–5.08 (m, 4H), 6.39–6.50 (m, 2H), 6.91–7.53 (m, 9H); δC (100.7 MHz, CDCl_3_): 62.4, 110.0, 110.2, 110.5, 110.6, 114.6, 115.8, 124.8, 127.8, 128.3, 129.7, 130.3, 130.4, 131.4, 133.9, 135.0, 137.3, 143.1, 143.3, 147.7, 148.0, 149.9, 150.1, 158.6, 158.8; IR ν_max_ (cm^−1^): 3,402 (w), 2,921 (w), 1,606 (m), 1,507 (s), 1,241 (s), 1,012 (m), 836 (s); HRMS: m/z calcd for C_18_H_16_ClN_2_O_2_: 327.0900; found: 327.0894 [M + H]^+^.

### Synthesis of 4-chlorophenyl-4′-furfuryloxyphenyl diazomethane

According to the references^28,44^, a mixture of manganese dioxide (2.52 g, 28.4 mmol), sodium sulfate (2.17 g, 15.3 mmol), and potassium hydroxide (0.86 g, 15.4 mmol) was added to a solution of **2** (1.72 g, 5.26 mmol) in dichloromethane (80 ml). The mixture was vigorously stirred for 10 h in the dark, and then filtered through a pad of Celite. The filtrate was concentrated under vacuum to yield **3** (98%) as a purple solid and stored at − 20 °C to avoid decomposition. m.p.: 78.5–80.4 °C; δH (400.3 MHz, CDCl_3_): 5.04 (s, 2H), 6.41–6.47 (dd, 2H), 7.05–7.14 (m, 4H), 7.24–7.34 (m, 4H), 7.48 (m, 1H); δC (100.7 MHz, CDCl_3_): 60.8, 62.1, 109.6, 110.1, 115.5, 120.4, 124.8, 127.1, 128.5, 128.7, 129.9, 142.7, 149.5, 156.7; IR ν_max_ (cm^−1^): 2,924 (w), 2,032 (vs), 1,600 (w), 1507 (s), 1,239 (s), 821 (m), 746 (m); HRMS: m/z calcd for C_18_H_14_ClN_2_O_2_: 325.0744; found: 325.0731 [M + H]^+^.

### Synthesis and modification of AgNPs

Polyvinylpyrrolidone (10.5 g) was added to glycol (49.8 ml), and the mixture was heated to 60 °C with magnetic stir to give a colorless transparent solution. AgNO_3_ (1.5 g) was then added. The mixture was heated to 120 °C and stirred for 1.5 h. Glycol was used as solvent and reductant, and polyvinylpyrrolidone was used as dispersant. The reaction mixture was cooled to room temperature to give Ag NPs dispersion. After the dispersion was centrifugated, the precipitate was washed by acetone (50 ml) for 3 times, and dried under vacuum to give Ag NPs powder.

Ag NPs (0.191 g) were ultrasonically dispersed in a solution of diazomethane **3** (47.7 mg) in dichloromethane (10 ml). The dispersion was evaporated carefully to absorb the diazomethane evenly on the surface of Ag NPs. The solid mixture was heated at 120 °C for 30 min, after which the color changed from purple to yellow. The residue was washed with dichloromethane by ultrasonic treatment, centrifuged (30 ml × 6), and dried under vacuum (rt., 24 h) to give furyl-modified Ag NPs.

### Controlled crosslink of AgNPs

The furyl-modified Ag NPs (0.195 g) were dispersed in dichloromethane (5 ml). To this was added a solution of *N*,*N*′-(4,4′-methylenediphenyl) dimaleimide (40.1 mg, 0.112 mmol) in dimethyl sulfoxide (9 ml). The mixture was heated at 50 °C, 70 °C, 90 °C, 110 °C, and 130 °C for 4 h, respectively, cooled to room temperature, and stored in the refrigerator.

For some related characterizations, the mixture was washed with dichloromethane by ultrasonic treatment, centrifuged (30 ml × 5), and dried under vacuum (45 °C, 24 h) to give a powder of crosslinked Ag NPs.

### Bioassay procedure

Hole-plate agar diffusion method was used to evaluate the antibacterial activity of aggregated Ag NP samples. Firstly, the bacterial solution (5 × 10^8^ CFU/ml) was prepared beforehand, mixed with molten agar, and pipetted into empty Petri dishes (90 mm) to form a 10 mm-thick agar layer. These Petri dishes with seeded agar were stored in the refrigerator until needed.

Secondly, the seeded agar plate was punched to give a 10 mm-diameter well, and each required Ag NPs sample (1.51 × 10^–3^ mg) with some water (~ 50 μl) was added to different well respectively. The well was then covered with some agar for encapsulation. After all the silver samples were loaded, the plates were incubated at 37 °C for 24 h to encourage bacterial growth. The diameter of the antimicrobial clear zones around each Ag NPs sample was measured and recorded.

Finally, an aq. solution of penicillin G potassium salt (1.0 mg ml^−1^) was used for calibration. Different amount solution was added to the pre-punched well of seeded agar plate, namely, 20 µl, 30 µl, 40 µl, and 50 µl for *E. coli*, 100 µl, 120 µl, 140 µl, and 160 µl for S. aureus. The agar plates were incubated for 24 h to encourage bacterial growth. The diameter of the antimicrobial clear zones around each well was measured and recorded.

## Supplementary information

Supplementary Information.

## Data Availability

The datasets generated and/or analyzed during the current study are available from the corresponding author on reasonable request.
