# Supplementary material for: Carbene modification and reversible crosslinking of silver nanoparticles for controlled antibacterial activity
Source: Sci Rep. 2020 Sep 10;10:14937. doi: 10.1038/s41598-020-72043-1 (PMC7484751; doi:10.1038/s41598-020-72043-1)
Supplement: Supplementary file 1 — Supplementary Information. [file 41598_2020_72043_MOESM1_ESM.docx]

Supplementary Materials

**Carbene modification and reversible crosslinking of silver nanoparticles for controlled antibacterial activity**

Liling Jing^a^, Mark G. Moloney^b,c^, Hao Xu^a^, Lian Liu^a^, Wenqiang Sun^a^, Junying Li^a^, Pengfei Yang^a,^*

*^a^School of Chemistry and Pharmaceutical Engineering, Qilu University of Technology (Shandong Academy of Sciences), Jinan 250353, PR China*

*^b^Chemistry Research Laboratory, Department of Chemistry, University of Oxford, Oxford OX1 3TA, UK*

*^c^Oxford Suzhou Centre for Advanced Research, Suzhou 215123, PR China*

****Corresponding author.*** Pengfei Yang. E-mail: ypf@qlu.edu.cn. Tel: +86-531-89631212. Fax: +86-531-89631212. ORCID: 0000-0003-4346-7367.

**1. Controlled release behavior determined by electroconductivity**

When Ag NPs were dispersed in water, Ag^+^ would be released continuously so as to enlarge its electroconductivity. Two typical dispersions were prepared by mixing 90°C-crosslinked Ag NPs and 130°C-crosslinked Ag NPs with distilled water (0.39 g·L^-1^), respectively. The electroconductivity of these dispersions was measured at room temperature and the results were shown in Figure S1. There was a stage of rapid release before 6 hours and then leveled off as time went on.


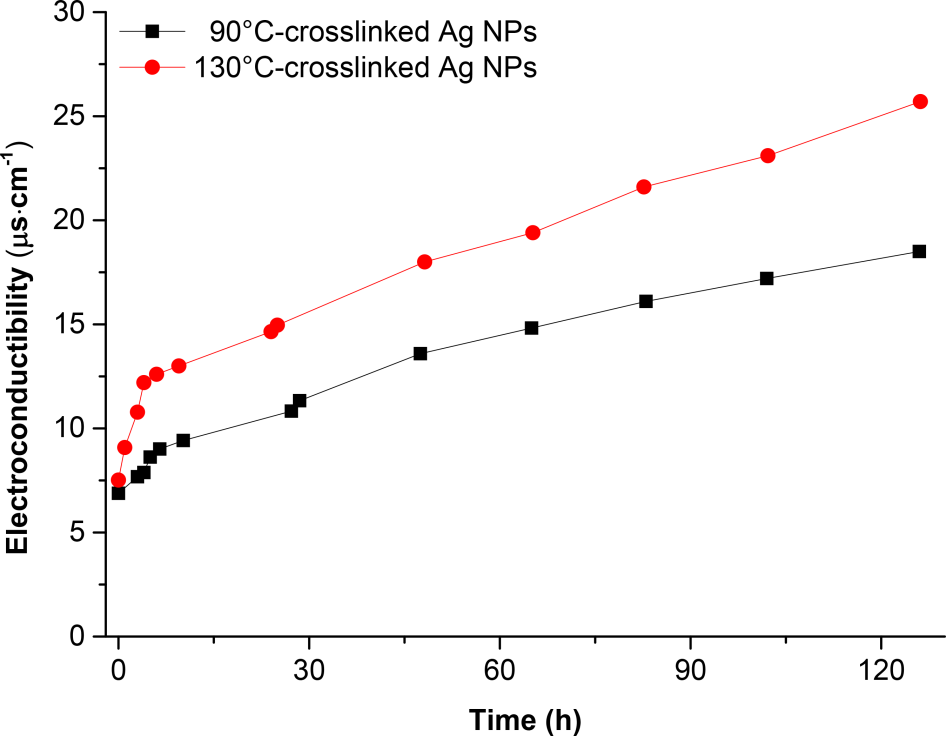


Figure S1 The evolution of electroconductivity for different Ag NPs dispersions

**2. UV-vis spectroscopy of Ag NPs**

The Ag NPs dispersion or the crosslinked Ag NPs dispersion was diluted with ethanol, and their volume ratio was 1:100 and 1:1000, respectively. Their UV-vis spectra were recorded and shown in Figure S2 and Figure S3. When Ag NPs dispersion was diluted by 100 times, a clear peak in the region of 430~440 nm was found, which was the characteristic peak of Ag NPs. When Ag NPs was diluted by 1000 times, another peak in the region of 280~290 nm appeared only for the crosslinked samples. The UV-vis curves seemed very similar in Figure S2 and S3 although Ag NPs were crosslinked at different temperature.


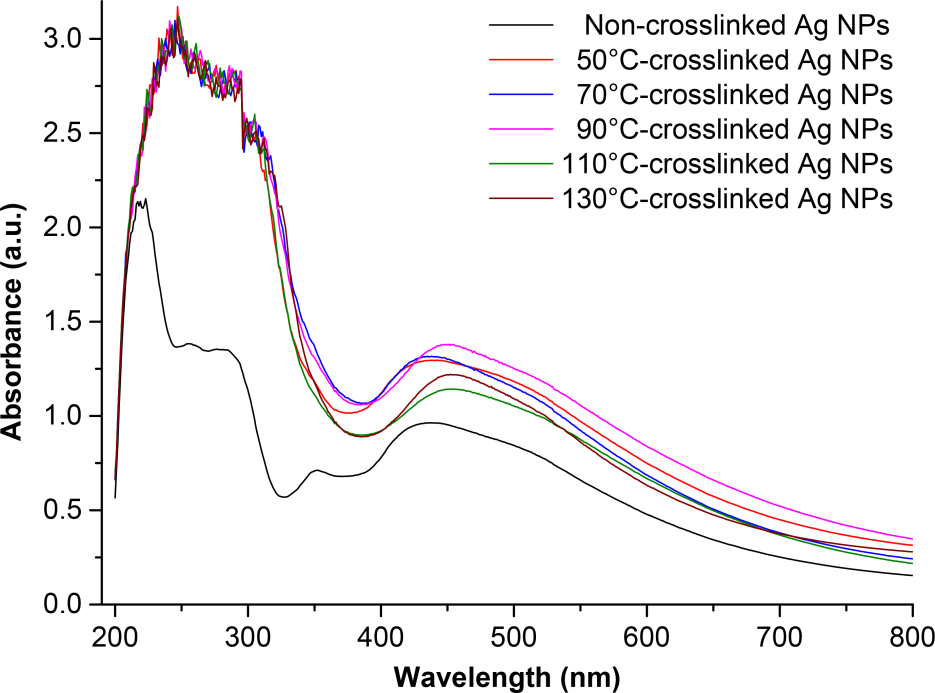


Figure S2 UV-vis spectra of Ag NPs dispersion (diluted by 100 times)


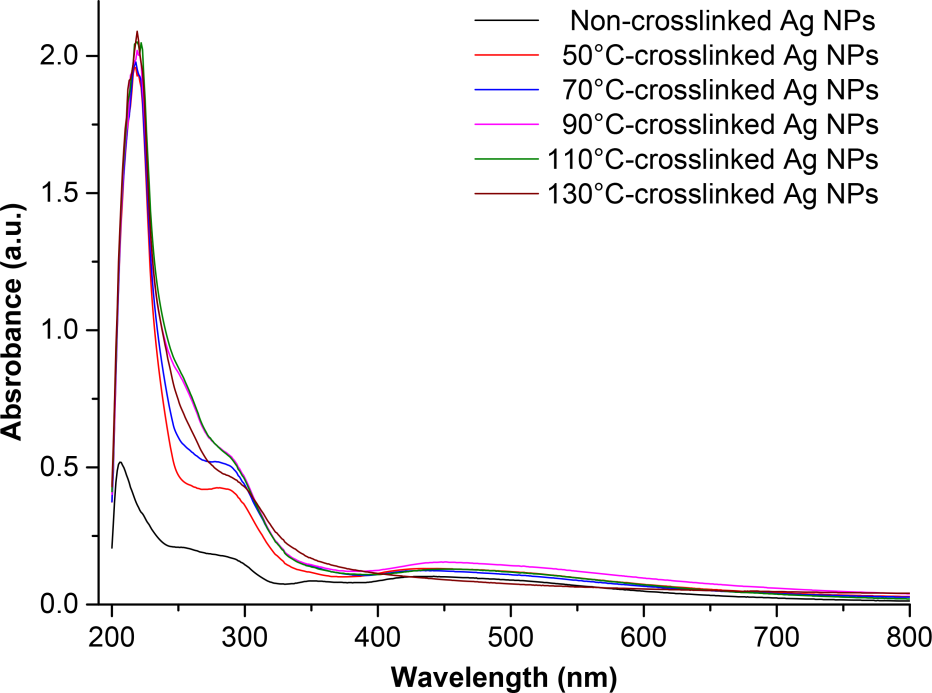


Figure S3 UV-vis spectra of Ag NPs dispersion (diluted by 1000 times)

**3. Typical photos of agar plate of antibacterial experiment**

The antibacterial activity of different Ag NPs was measured by inhibition zone method. Each sample was tested for three times to give an average value for accuracy. The photo of non-crosslinked Ag NPs against *Escherichia coli* and *Staphylococcus aureus* were shown in Figure S4 and Figure S5. Clear inhibition zones were found in the picture.


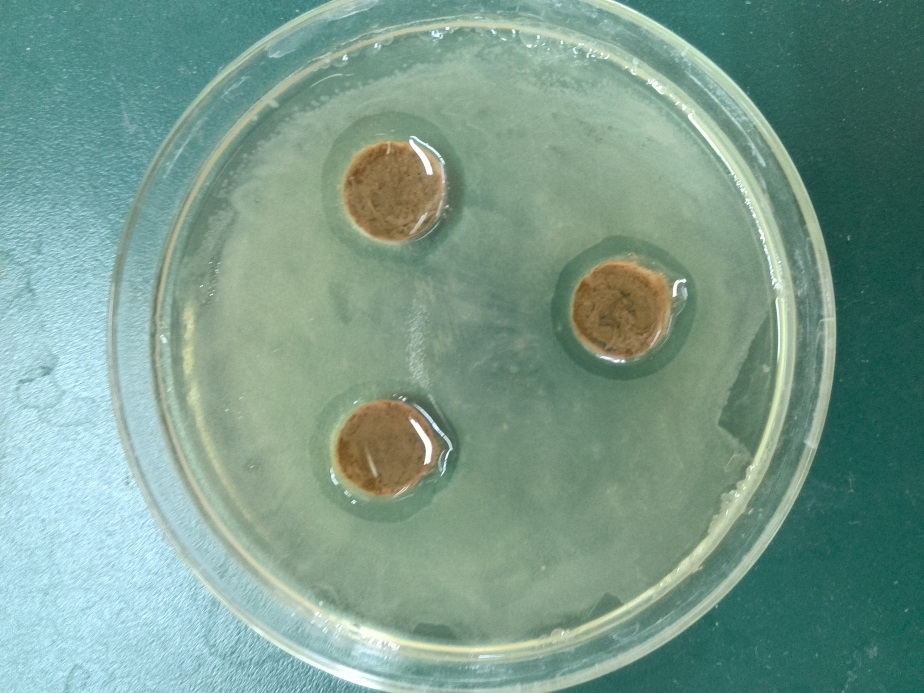


Figure S4 Antibacterial activity of non-crosslinked Ag NPs against *Escherichia coli* (parallel experiments)


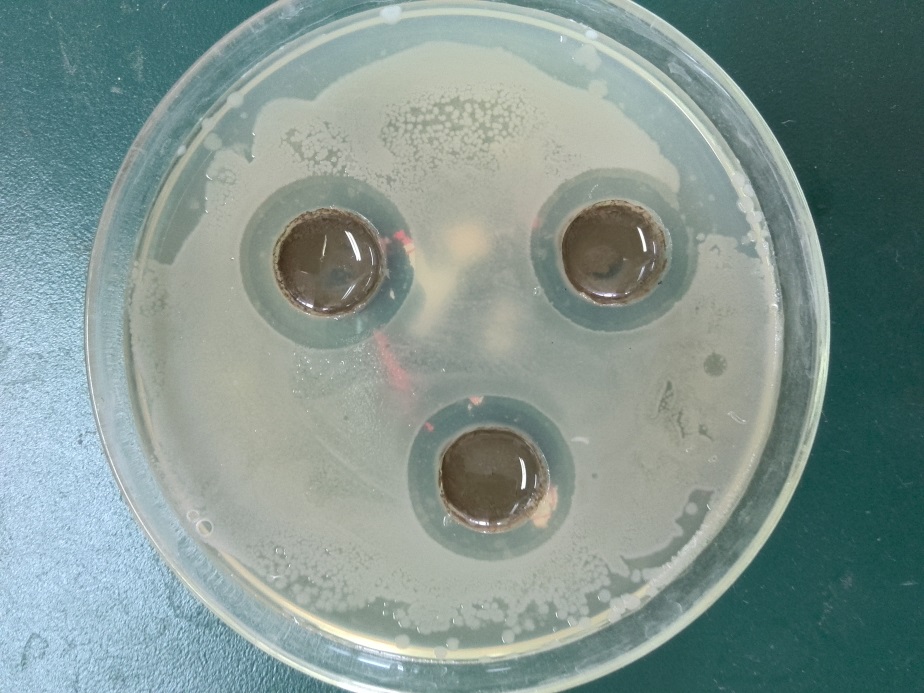


Figure S5 Antibacterial activity of non-crosslinked Ag NPs against *Staphylococcus aureus* (parallel experiments)

**4. NMR, IR, and MS spectra of compounds**





Figure S6 ^1^H NMR spectra of **1**





Figure S7 ^13^C NMR spectra of **1**





Figure S8 IR spectra of **1**





Figure S9 MS spectra of **1**





Figure S10 ^1^H NMR spectra of **2**





Figure S11 ^13^C NMR spectra of **2**





Figure S12 IR spectra of **2**





Figure S13 MS spectra of **2**





Figure S14 ^1^H NMR spectra of **3**





Figure S15 ^13^C NMR spectra of **3**





Figure S16 IR spectra of **3**





Figure S17 MS spectra of **3**
